# Frontiers of monkeypox research: An analysis from the top 100 most influential articles in the field^☆^

**DOI:** 10.1016/j.heliyon.2023.e20566

**Published:** 2023-10-02

**Authors:** Xuhao Li, Yang Li, Wenyan Yu, Zhixia Jia, Jinling Li, Yuanxiang Liu, Jiguo Yang

**Affiliations:** a School of Acupuncture-Moxibustion and Tuina, Shandong University of Traditional Chinese Medicine, Jinan 250355, China; b The First Clinical Medical College, Shandong University of Traditional Chinese Medicine, Jinan 250355, China

**Keywords:** Monkeypox, Bibliometric analysis, Top 100 cited papers, Trends, citeSpace, VOSviewer, Scimago graphica

## Abstract

**Background:**

Monkeypox (MPX) has made recurrence after decades as a neglected zoonotic disease. More nations have reported endemic monkeypox in the past decade than in the previous forty. The World Health Organization has warned that the world may face another significant challenge after dealing with COVID-19, a pandemic, and the Monkeypox outbreak. Early appraisal of monkeypox research and development allows researchers to anticipate solutions for large outbreaks. We conducted a bibliometric analysis of this study's top 100 cited papers to identify regional research patterns.

**Methods:**

Our method was to search the SCI-Expanded database on Web of Science (WOS) for the top 100 papers that were cited in MPX on this database. We examined relevant literature from different years, journals, countries/regions, institutions, authors, and keywords.In order to create knowledge maps, we used the programs VOSviewer, Citespace, Scimago Graphica and the bibliometric online analysis platform. After compiling the relevant literature in Excel, we could estimate the field's focus and trends.

**Results:**

A total of 47 journals from 36 countries and regions published the top 100 cited papers between 1999 and 2023. The majority of articles were published in *EMERGING INFECTIOUS DISEASES*, while the highest average number of citations per paper were found in the *NEW ENGLAND JOURNAL OF MEDICINE.* The UNITED STATES contributed the most publications, followed by ENGLAND and SWITZERLAND. As far as the total number of publications goes, the Centers for Disease Control & Prevention in the USA, the National Institute of Health in the USA, and the World Health Organization each contributed the most papers. The major categories are immunology, virology and infectious diseases. The top five keywords were infection, Congo, virus, smallpox, and transmission. The cluster analysis suggests MPX research will focus on safe and effective vaccines and epidemic prevention.

**Conclusion:**

By using bibliometric analysis, MPX researchers can quickly and visually identify their research focus and boundaries. Although studies suggest that antiviral medicine is the best treatment, creating an effective vaccine might lessen and avoid MPX pandemics in the long term. Our findings imply that safe and effective vaccines may be the focus and trends for future MPX research. International coordination for case monitoring and identification is essential to understand monkeypox disease's ever-changing epidemiology.

## 1 Introduction

Monkeypox virus (MPXV) is a double-helix DNA virus that belongs to the Orthopoxvirus genus of the Poxviridae family. Monkeypox (MPX) disease is brought on by the Poxviridae subfamily of the Chordopoxvirinae and Orthopoxvirus genera [1,2]. Contact with infected animals/people, respiratory droplets, and vertical mother-to-child transmission are all ways for the MPXV to spread [3]. Despite its less clinical severity, MPX is a disease with similar symptoms to smallpox [4]. Most immune-competent individuals can deal with vaccinia and cowpox infections, but MPX infections can be fatal and severe [5]. The global eradication of smallpox led MPX to become one of the most serious orthopoxvirus infections [6]. A higher risk of mortality and debilitating disease is seen in individuals with impaired immune function.

In 1959, MPXV was discovered as an illness affecting monkeys at a research institute in Copenhagen, Denmark [7]. The first human MPX case in 1970 occurred in the Demographic Republic of Congo, which has since become the center of poverty-related MPX cases in East and Central Africa [8]. In 2003, MPX was reported in the United States for first-time outside Africa [9]. MPX has emerged in non-endemic regions in 2022, despite the global COVID-19 pandemic still ongoing. This presents a new global threat [10]. As of November 16, 2022, 110 countries had reported 79411 laboratory confirmed MPX cases and 50 fatalities to WHO [11].

Due to virology, epidemiology, and clinical diagnosis variations, MPX has been divided into two genetic clades: West African and Central African [12]. MPXV typically takes 6–13 days to incubate, with the longest cases lasting 21 days. The investigation confirmed that the case fatality rate of patients infected with central and non-branch MPXV was 10.6% [11,13,14]. In July 2022, the WHO proclaimed MPX an international public health emergency (15). MPX usually starts with a localized rash and later spreads (16).

Researchers have made significant progress in MPX diagnosis and treatment in recent years [15,16]. Yet, it is challenging to understand the overall advancements and research trends in MPX disease [17,18]. In MPX fields, bibliometric analysis is an important part of the evaluation process. The top 100 MPX publications will give the researcher a better understanding of the current study topic. Bibliometric analysis may assist researchers adequately in a certain current research field and is an extremely effective way of bibliometric analysis [19,20]. Many specialties and sub-specialties have been analyzed using bibliometric methods, but we found no about MPX in our literature review. After obtaining the top 100 cited MPX publications, we used bibliometric analysis to assess MPX research focus and trends. We used bibliometric analysis to identify MPX research trends and anticipate hotspots.

## 2 Materials and methods

### 2.1 Data sources and search strategies

A thorough search of the Web of Science (WOS) Core Collection database was conducted on February 28, 2023, to find the top 100 cited MPX articles published between 1999 and 2023 (https://www.webofscience.com/wos/woscc/basic-search). The following was the framework for the literature search: TI = ("Monkeypox virus*") OR TI = ("Monkeypox*") OR TI = ("Monkey pox virus*") OR TI = ("Monkey pox*") OR TI = ("Mpox*") OR TI = ("Mpox virus*"). The publication category was restricted to English-language articles and reviews.

### 2.2 Inclusion and exclusion criteria

In order of decreasing citation frequency, publications were graded in order of increasing citation frequency.After eliminating non-MPX-related papers, the titles, abstracts, and full texts of the papers were examined to determine the 100 papers with the highest number of citations. A third researcher was contacted when two researchers could not agree on an article's inclusion.

### 2.3 Data collection

Following the import of the selected articles into NoteExpress, key study parameters, such as title, number of citations, keywords, publication years, authors, affiliations, paper type, references, countries/regions, and journals, were extracted.We will import the parameters and data obtained from WOS in "Txt" format into the visualization analysis software for further analysis. During this process, researchers will correct any erroneous words and merge overlapping terms. During this meticulous process, data accuracy and integrity are ensured, allowing it to be analyzed and researched further.

### 2.4 Bibliometric analysis

We employed CiteSpace 6.1.R6, VOSviewer 1.6.19, and Scimago Graphica 1.0.30, and the bibliometric online analysis platform (https://bibliometric.com) to visualize and analyze the characteristics of articles in order to identify the present research focus and potential trends in the MPX field. The study used VOSviewer to construct scientometric networks and visualize knowledge [21]. Using CiteSapce, one can gain a better understanding of MPX hotspot bursts [22].

## 3 Results

### 3.1 Citation characteristics of the included

Articles.

**Fig. 1:**
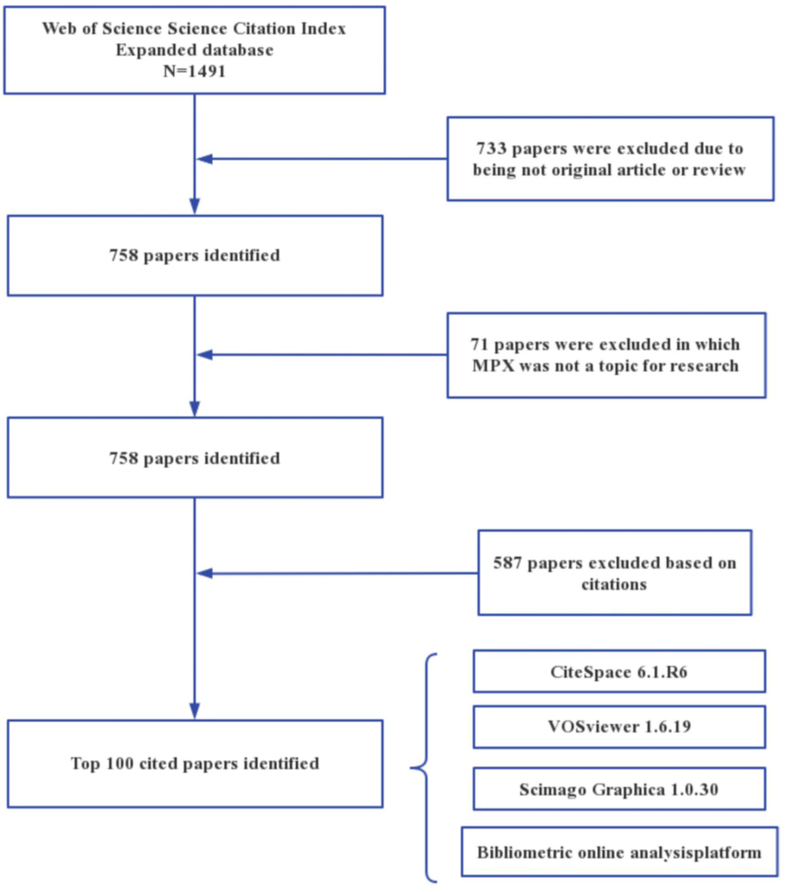
Flowchart of literature screening.

Until February 28, 2023, 1491 MPX studies had been published, with 758 papers not being articles or reviews.Following exclusion of studies not related to MPX, 687 studies were left.Eligible articles were ordered descendingly by their frequency of citations. Fig. 1 illustrates the flow diagram for the specific process.

Table 1 lists the top 100 cited papers with a total number of 12,065 citations (mean = 12.06). The top 100 cited papers contained 3918 references. The most frequently cited articles included "The detection of monkeypox in humans in the Western Hemisphere" [23] by Reed, KD (480 citations), followed by "Monkeypox Virus Infection in Humans across 16 Countries - April–June 2022" (345 citations) [24], "Major increase in human monkeypox incidence 30 years after smallpox vaccination campaigns cease in the Democratic Republic of Congo" (314 citations) [25]. Over 300 citations have been made to the articles mentioned above.

**Table 1:** MPX's top 100 cited papers until 2023.

Rank	Authors	Title	Journal	Totalcitation	Publication Year	Average citationby year
1	Reed, KD	The detection of monkeypox in humans in the Western Hemisphere	NEW ENGLAND JOURNAL OF MEDICINE	496	2004	26.11
2	Thornhill, JP	Monkeypox Virus Infection in Humans across 16 Countries - April–June 2022	LANCET NEUROLOGY	406	2022	406.00
3	Rimoin, AW	Major increase in human monkeypox incidence 30 years after smallpox vaccination campaigns cease in the Democratic Republic of Congo	PROCEEDINGS OF THE NATIONAL ACADEMY OF SCIENCES OF THE UNITED STATES OF AMERICA	326	2010	25.08
4	Earl, PL	Human monkeypox: an emerging zoonosis	LANCET INFECTIOUS DISEASES	303	2004	15.95
5	Likos, AM	A tale of two clades: monkeypox viruses	JOURNAL OF GENERAL VIROLOGY	300	2005	16.67
6	Di Giulio, DB	Human Monkeypox	CLINICAL INFECTIOUS DISEASES	299	2014	33.22
7	Rogers, JV	Immunogenicity of a highly attenuated MVA smallpox vaccine and protection against monkeypox	NATURE	299	2004	15.74
8	McCollum, AM	A preliminary assessment of silver nanoparticle inhibition of monkeypox virus plaque formation	NANOSCALE RESEARCH LETTERS	285	2008	19.00
9	Edghill-Smith, Y	Outbreak of human monkeypox, Democratic Republic of Congo, 1996–1997	EMERGING INFECTIOUS DISEASES	247	2001	11.23
10	Hutin, YJF	Smallpox vaccine-induced antibodies are necessary and sufficient for protection against monkeypox virus	NATURE MEDICINE	241	2005	13.39
11	Huhn, GD	Clinical characteristics of human monkeypox, and risk factors for severe disease	CLINICAL INFECTIOUS DISEASES	219	2005	12.17
12	Chen, NH	Outbreak of human monkeypox in Nigeria in 2017-18: a clinical and epidemiological report	LANCET INFECTIOUS DISEASES	217	2019	54.25
13	Yinka-Ogunleye, A	Virulence differences between monkeypox virus isolates from West Africa and the Congo basin	VIROLOGY	205	2005	11.39
14	Hooper, JW	A systematic review of the epidemiology of human monkeypox outbreaks and implications for outbreak strategy	PLOS NEGLECTED TROPICAL DISEASES	198	2019	49.50
15	Beer, EM	Smallpox DNA vaccine protects nonhuman primates against lethal monkeypox	JOURNAL OF VIROLOGY	191	2004	10.05
16	Learned, LA	Monkeypox Virus in Nigeria: Infection Biology, Epidemiology, and Evolution	VIRUSES-BASEL	184	2020	61.33
17	Alakunle, E	Epidemiological, clinical and virological characteristics of four cases of monkeypox support transmission through sexual contact, Italy, May 2022	EUROSURVEILLANCE	183	2022	183.00
18	Durski, KN	Emergence of Monkeypox as the Most Important Orthopoxvirus Infection in Humans	FRONTIERS IN PUBLIC HEALTH	180	2018	36.00
19	Sklenovska, N	Emergence of Monkeypox - West and Central Africa, 1970–2017	MMWR-MORBIDITY AND MORTALITY WEEKLY REPORT	177	2018	35.40
20	Parker, S	Extended interhuman transmission of monkeypox in a hospital community in the Republic of the Congo, 2003	AMERICAN JOURNAL OF TROPICAL MEDICINE AND HYGIENE	177	2005	9.83
21	Antinori, A	Clinical manifestations of human monkeypox influenced by route of infection	JOURNAL OF INFECTIOUS DISEASES	172	2006	10.12
22	Zaucha, GM	Human monkeypox: an emerging zoonotic disease	FUTURE MICROBIOLOGY	167	2007	10.44
23	Reynolds, MG	The pathology of experimental aerosolized monkeypox virus infection in cynomolgus monkeys (Macaca fascicularis)	LABORATORY INVESTIGATION	167	2001	7.59
24	Baker, R	Potential antiviral therapeutics for smallpox, monkeypox and other orthopoxvirus infections	ANTIVIRAL RESEARCH	164	2003	8.20
25	Li, Y	Human Monkeypox Epidemiologic and Clinical Characteristics, Diagnosis, and Prevention	INFECTIOUS DISEASE CLINICS OF NORTH AMERICA	161	2019	40.25
26	Petersen, E	Detection of monkeypox virus with real-time PCR assays	JOURNAL OF CLINICAL VIROLOGY	159	2006	9.35
27	Stittelaar, KJ	Two cases of monkeypox imported to the United Kingdom, September 2018	EUROSURVEILLANCE	154	2018	30.80
28	Isidro, J	Phylogenomic characterization and signs of microevolution in the 2022 multi-country outbreak of monkeypox virus	NATURE MEDICINE	153	2022	153.00
29	Vaughan, A	Modified vaccinia virus Ankara protects macaques against respiratory challenge with monkeypox virus	JOURNAL OF VIROLOGY	148	2005	8.22
30	Meyer, H	Diagnosis of Imported Monkeypox, Israel, 2018	EMERGING INFECTIOUS DISEASES	140	2019	35.00
31	Stittelaar, KJ	Human-to-Human Transmission of Monkeypox Virus, United Kingdom, October 2018	EMERGING INFECTIOUS DISEASES	135	2020	45.00
32	Erez, N	Outbreaks of disease suspected of being due to human monkeypox virus infection in the Democratic Republic of Congo in 2001	JOURNAL OF CLINICAL MICROBIOLOGY	135	2002	6.43
33	Guarner, J	Human monkeypox and smallpox viruses: genomic comparison	FEBS LETTERS	134	2001	6.09
34	Shchelkunov, SN	Reemergence of monkeypox: Prevalence, diagnostics, and countermeasures	CLINICAL INFECTIOUS DISEASES	133	2005	7.39
35	Nalca, A	Antiviral treatment is more effective than smallpox vaccination upon lethal monkeypox virus infection	NATURE	130	2006	.65
36	Hammarlund, E	Monkeypox transmission and pathogenesis in prairie dogs	EMERGING INFECTIOUS DISEASES	130	2004	6.84
37	Vaughan, A	Real-time PCR assays for the specific detection of monkeypox virus West African and Congo Basin strain DNA	JOURNAL OF VIROLOGICAL METHODS	129	2010	9.92
38	Li, Y	Clinical features and novel presentations of human monkeypox in a central London centre during the 2022 outbreak: descriptive case series	BMJ-BRITISH MEDICAL JOURNAL	123	2022	123.00
39	Patel, A	Multiple diagnostic techniques identify previously vaccinated individuals with protective immunity against monkeypox	NATURE MEDICINE	123	2005	6.83
40	Karem, KL	Prevention and Treatment of Monkeypox	DRUGS	112	2022	112.00
41	Rizk, JG	Community transmission of monkeypox in the United Kingdom, April to May 2022	EUROSURVEILLANCE	110	2022	110.00
42	Vivancos, R	Extended Human-to-Human Transmission during a Monkeypox Outbreak in the Democratic Republic of the Congo	EMERGING INFECTIOUS DISEASES	109	2016	15.57
43	Simpson, K	Human monkeypox - After 40 years, an unintended consequence of smallpox eradication	VACCINE	108	2020	36.00
44	Nolen, LD	Clinical presentation and virological assessment of confirmed human monkeypox virus cases in Spain: a prospective observational cohort study	LANCET	106	2022	106.00
45	Huggins, J	Characterization of acute-phase humoral immunity to monkeypox: Use of immunoglobulin M enzyme-linked immunosorbent assay for detection of monkeypox infection during the 2003 North American outbreak	CLINICAL AND DIAGNOSTIC LABORATORY IMMUNOLOGY	105	2005	5.83
46	Hutson, CL	Maternal and Fetal Outcomes Among Pregnant Women With Human Monkeypox Infection in the Democratic Republic of Congo	JOURNAL OF INFECTIOUS DISEASES	103	2017	17.17
47	Shchelkunov, SN	Human monkeypox infection: A family cluster in the Midwestern United States	JOURNAL OF INFECTIOUS DISEASES	103	2004	5.42
48	Mbala, PK	Analysis of the monkeypox virus genome	VIROLOGY	103	2002	4.90
49	Heraud, JM	Genomic Variability of Monkeypox Virus among Humans, Democratic Republic of the Congo	EMERGING INFECTIOUS DISEASES	101	2014	11.22
50	Sejvar, JJ	Nonhuman Primates Are Protected from Smallpox Virus or Monkeypox Virus Challenges by the Antiviral Drug ST-246	ANTIMICROBIAL AGENTS AND CHEMOTHERAPY	101	2009	7.21
51	Hutson, CL	Monkeypox zoonotic associations: Insights from laboratory evaluation of animals associated with the multi-state us outbreak	AMERICAN JOURNAL OF TROPICAL MEDICINE AND HYGIENE	101	2007	6.31
52	Minhaj, FS	Subunit recombinant vaccine protects against monkeypox	JOURNAL OF IMMUNOLOGY	98	2006	5.76
53	Ogoina, D	Frequent detection of monkeypox virus DNA in saliva, semen, and other clinical samples from 12 patients, Barcelona, Spain, May to June 2022	EUROSURVEILLANCE	97	2022	97.00
54	Kugelman, JR	Ongoing monkeypox virus outbreak, Portugal, 29 April to May 23, 2022	EUROSURVEILLANCE	97	2022	97.00
55	Duque, MP	The 2017 human monkeypox outbreak in Nigeria-Report of outbreak experience and response in the Niger Delta University Teaching Hospital, Bayelsa State, Nigeria	PLOS ONE	97	2019	24.25
56	Rao, AK	Monkeypox Outbreak - Nine States, May 2022	MMWR-MORBIDITY AND MORTALITY WEEKLY REPORT	96	2022	96.00
57	Mauldin, MR	Exportation of Monkeypox Virus From the African Continent	JOURNAL OF INFECTIOUS DISEASES	96	2022	96.00
58	Yong, SEF	Monkeypox in a Traveler Returning from Nigeria - Dallas, Texas, July 2021	MMWR-MORBIDITY AND MORTALITY WEEKLY REPORT	95	2022	95.00
59	Damon, IK	Imported Monkeypox, Singapore	EMERGING INFECTIOUS DISEASES	94	2020	31.33
60	Smee, DF	Status of human monkeypox: clinical disease, epidemiology and research	VACCINE	94	2011	7.83
61	Reynolds, MG	A prairie dog animal model of systemic orthopoxvirus disease using West African and Congo Basin strains of monkeypox virus	JOURNAL OF GENERAL VIROLOGY	94	2009	6.71
62	Peiro-Mestres, A	Outbreaks of human monkeypox after cessation of smallpox vaccination	TRENDS IN MICROBIOLOGY	89	2012	8.09
63	Tarin-Vicente, EJ	Characterization of wild-type and cidofovir-resistant strains of camelpox, cowpox, monkeypox, and vaccinia viruses	ANTIMICROBIAL AGENTS AND CHEMOTHERAPY	85	2002	4.05
64	Bragazzi, NL	Epidemiological trends and clinical features of the ongoing monkeypox epidemic: A preliminary pooled data analysis and literature review	JOURNAL OF MEDICAL VIROLOGY	84	2023	84
65	Earl, PL	Human Monkeypox Outbreak Caused by Novel Virus Belonging to Congo Basin Clade, Sudan, 2005	EMERGING INFECTIOUS DISEASES	84	2010	6.46
66	Liszewski, MK	Endemic human monkeypox, democratic Republic of Congo, 2001–2004	EMERGING INFECTIOUS DISEASES	83	2007	5.19
67	Parker, S	Reemergence of Human Monkeypox in Nigeria, 2017	EMERGING INFECTIOUS DISEASES	82	2018	16.40
68	Formenty, P	A review of experimental and natural infections of animals with monkeypox virus between 1958 and 2012	FUTURE VIROLOGY	82	2013	8.20
69	Rimoin, AW	Monkeypox virus and insights into its immunomodulatory proteins	IMMUNOLOGICAL REVIEWS	81	2008	5.40
70	Radonic, A	Rapid protection in a monkeypox model by a single injection of a replication-deficient vaccinia virus	PROCEEDINGS OF THE NATIONAL ACADEMY OF SCIENCES OF THE UNITED STATES OF AMERICA	81	2008	5.40
71	Weaver, JR	Structure and regulatory profile of the monkeypox inhibitor of complement: Comparison to homologs in vaccinia and variola and evidence for dimer formation	JOURNAL OF IMMUNOLOGY	81	2006	4.76
72	Jordan, R	Fatal Monkeypox in Wild-Living Sooty Mangabey, Cole d'Ivoire, 2012	EMERGING INFECTIOUS DISEASES	79	2014	8.78
73	Levine, RS	Family cluster of three cases of monkeypox imported from Nigeria to the United Kingdom, May 2021	EUROSURVEILLANCE	78	2021	39.00
74	Jin, YH	Demographic and clinical characteristics of confirmed human monkeypox virus cases in individuals attending a sexual health centre in London, UK: an observational analysis	LANCET INFECTIOUS DISEASES	76	2022	76.00
75	Yinka-Ogunleye, A	The 2022 outbreak and the pathobiology of the monkeypox virus	JOURNAL OF AUTOIMMUNITY	76	2022	76.00
76	Saijo, M	ST-246 Antiviral Efficacy in a Nonhuman Primate Monkeypox Model: Determination of the Minimal Effective Dose and Human Dose Justification	ANTIMICROBIAL AGENTS AND CHEMOTHERAPY	75	2009	5.36
77	Kumar, N	Spectrum of infection and risk factors for human monkeypox, United States, 2003	EMERGING INFECTIOUS DISEASES	75	2007	4.69
78	Tesh, RB	LC16m8, a highly attenuated vaccinia virus vaccine lacking expression of the membrane protein B5R, protects monkeys from monkeypox	JOURNAL OF VIROLOGY	75	2006	4.41
79	Reynolds, MG	Ecological Niche and Geographic Distribution of Human Monkeypox in Africa	PLOS ONE	74	2007	4.63
80	Hobson, G	Practical synthesis of D- and L-2-cyclopentenone and their utility for the synthesis of carbocyclic antiviral nucleosides against orthopox viruses (smallpox, monkeypox, and cowpox virus)	JOURNAL OF ORGANIC CHEMISTRY	74	2003	3.70
81	Xiao, SY	Experimental infection of ground squirrels (Spermophilius tridecemlineatus) with Monkeypox virus	EMERGING INFECTIOUS DISEASES	73	2004	3.84
82	Doty, JB	Imported Monkeypox from International Traveler, Maryland, USA, 2021	EMERGING INFECTIOUS DISEASES	70	2022	70.00
83	Costello, V	Assessing Monkeypox Virus Prevalence in Small Mammals at the Human-Animal Interface in the Democratic Republic of the Congo	VIRUSES-BASEL	67	2017	11.17
84	Stabenow, J	Experimental infection of prairie dogs with monkeypox virus	EMERGING INFECTIOUS DISEASES	66	2005	3.67
85	Edghill-Smith, Y	Reemergence of Human Monkeypox and Declining Population Immunity in the Context of Urbanization, Nigeria, 2017–2020	EMERGING INFECTIOUS DISEASES	64	2021	32.00
86	Girometti, N	A Mouse Model of Lethal Infection for Evaluating Prophylactics and Therapeutics against Monkeypox Virus	JOURNAL OF VIROLOGY	62	2010	4.77
87	Reeves, PM	Smallpox vaccine does not protect Macaques with AIDS from a lethal Monkeypox virus challenge	JOURNAL OF INFECTIOUS DISEASES	62	2005	3.44
88	Hirao, LA	Multivalent Smallpox DNA Vaccine Delivered by Intradermal Electroporation Drives Protective Immunity in Nonhuman Primates Against Lethal Monkeypox Challenge	JOURNAL OF INFECTIOUS DISEASES	61	2011	5.08
89	Nguyen, PY	Variola and Monkeypox Viruses Utilize Conserved Mechanisms of Virion Motility and Release That Depend on Abl and Src Family Tyrosine Kinases	JOURNAL OF VIROLOGY	61	2011	5.08
90	Reynolds, MG	Pharmacokinetics and Efficacy of a Potential Smallpox Therapeutic, Brincidofovir, in a Lethal Monkeypox Virus Animal Model	MSPHERE	60	2021	30.00
91	Karem, KL	Monkeypox re-emergence in Africa: a call to expand the concept and practice of One Health	EXPERT REVIEW OF ANTI-INFECTIVE THERAPY	60	2019	15.00
92	Hutson, CL	Monkeypox infection presenting as genital rash, Australia, May 2022	EUROSURVEILLANCE	59	2022	59.00
93	Petersen, BW	Vaccinating against monkeypox in the Democratic Republic of the Congo	ANTIVIRAL RESEARCH	59	2019	14.75
94	Reynolds, MG	Monkeypox-induced immunity and failure of childhood smallpox vaccination to provide complete protection	CLINICAL AND VACCINE IMMUNOLOGY	59	2007	3.69
95	Americo, JL	Improving the Care and Treatment of Monkeypox Patients in Low-Resource Settings: Applying Evidence from Contemporary Biomedical and Smallpox Biodefense Research	VIRUSES-BASEL	58	2017	9.67
96	Manes, NP	Comparative proteomics of human monkeypox and vaccinia intracellular mature and extracellular enveloped virions	JOURNAL OF PROTEOME RESEARCH	55	2008	3.67
97	Hammerschlag, Y	Factors affecting the likelihood of monkeypox's emergence and spread in the post-smallpox era	CURRENT OPINION IN VIROLOGY	54	2012	4.91
98	Pippa, N	Identification of Wild-Derived Inbred Mouse Strains Highly Susceptible to Monkeypox Virus Infection for Use as Small Animal Models	JOURNAL OF VIROLOGY	53	2010	4.08
99	Reynolds, MG	DPPC: MPOx chimeric advanced Drug Delivery nano Systems (chi-aDDnSs): Physicochemical and structural characterization, stability and drug release studies	INTERNATIONAL JOURNAL OF PHARMACEUTICS	52	2013	5.20
100	Osorio, JE	Comparison of Monkeypox Viruses Pathogenesis in Mice by In Vivo Imaging	PLOS ONE	51	2009	3.64

### 3.2 Year of publication and citation

**Fig. 2:**
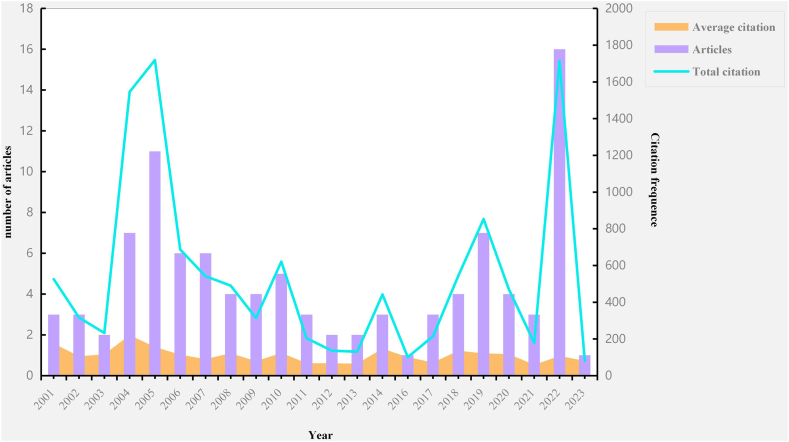
Fig.2 MPX publication and citation distribution by year.

On Fig. 2, we depicted the yearly citation frequency and the overall distribution of citations. Between 2001 and 2023, the 100 most cited papers were published. Articles published in 2015, on the other hand, did not fall within this range. It is important to note that 2004 had the highest average number of citations, while 2022 had the most published papers (N = 16). MPX research was greatly impacted by the publication in 2022 that had the greatest number of citations.

### 3.3 Distribution per journal

**Fig. 3:**
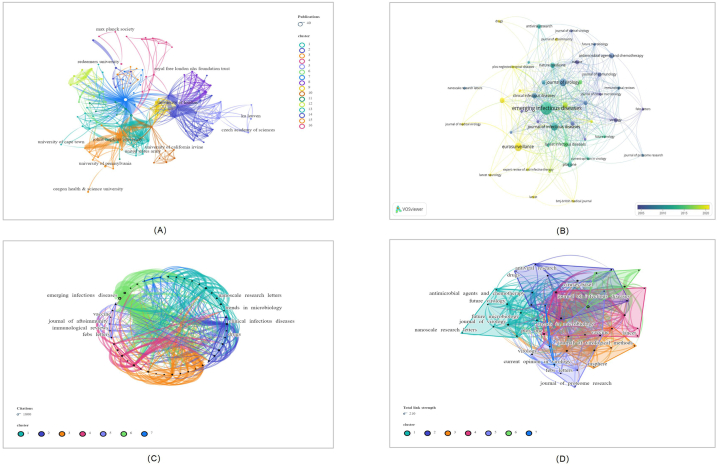
Visualization of the journals that contributed to the top 100 cited papers (Listed below are: A: The total number of publications; B: the number of publications that change annually; C: the total number of citations; D: Strength of total correlation. There are more individual units on a larger graph area, and the thicker the lines between two units, the stronger the correlation.).

The top 100 most cited papers were published by 47 journals. There were more publications in *EMERGING INFECTIOUS DISEASES* than any other journal(N = 16), followed by *EUROSURVEILLANCE* (N = 7)(Fig. 3A). Fig. 3B displays the annual publication count of articles by the journal. In terms of citations, *EMERGING INFECTIOUS DISEASES* received the greatest number (N = 1632). Contrarily, the NEW ENGLAND JOURNAL OF MEDICINE had the most citations per publication on average (1 paper, 496 citations). This figure displays the journals and citations that have been productive in the MPX field(Fig. 3C). Fig. 3D represents the total link strength of the association between journals. MMWR-MORBIDITY AND MORTALITY WEEKLY REPORT, EUROSURVEILLANCE, and LANCET NEUROLOGY have the most citations in the last three years.

**Fig. 4:**
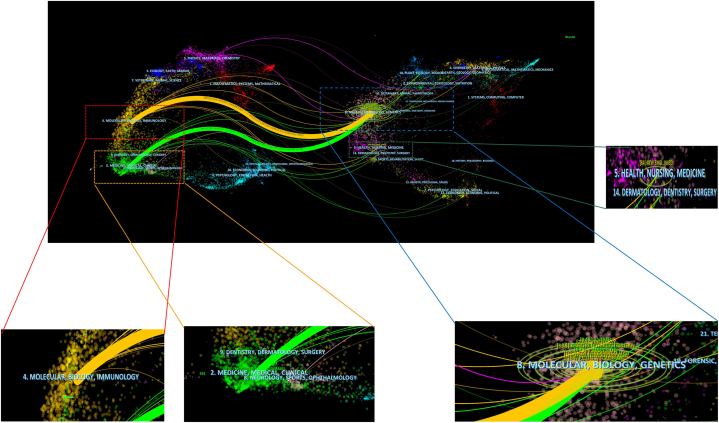
The MPX field has been overlayed with a dual map from CiteSpace.

In Fig. 4, we observed two major citation pathways associated with dual journal maps. Papers published in Molecular/Biology/Immunology, Medicine/Medical/Clinical or Neurology/Sports/Ophthalmology journals cited papers published in Molecular/Biology/Genetics, and Health/Nursing/Medicine, according to the two routes. This conclusion guided new MPX researchers.

### 3.4 Author and co-author analysis

**Fig. 5:**
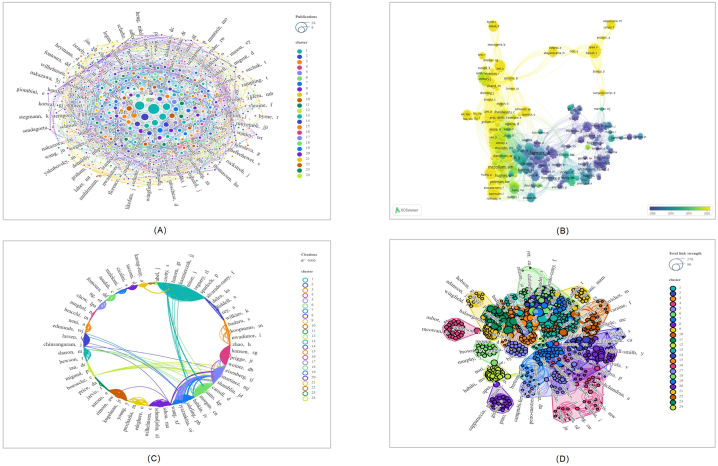
Visualization of the authors contributing to the top 100 cited papers (Listed below are: A: The total number of publications; B: the number of publications that change annually; C: the total number of citations; D: Strength of total correlation. There are more individual units on a larger graph area, and the thicker the lines between two units, the stronger the correlation.).

There was active cooperation amongst some of the author clusters of 13 distinct hues. Authors from 960 different institutions contributed to the top 100 papers. Inconsistent author names have been modified, like "Abel, JA" turning "Abel, J". Damon, IK authored the greatest number of papers (N = 24), followed by Reynolds, MG (N = 17). With Damon, IK as its core, the network has 269 connections, with 197 objects forming the largest group. (Fig. 5A). Fig. 5B displays the annual publication count of articles by authors. The authors with the most citations were Damon, IK (N = 3617), followed by Reynolds, MG (N = 2167) and Li, Y (N = 1868). However, Graham, MB, averaged the most citations per paper (1 paper, 496 citations) (Fig. 5C). Damon, IK is a leading writer, whereas Mccollum, AM and Ihekweazu, C are emerging writers (Fig. 5B). Fig. 5D represents the total link strength of the association among authors.

### 3.5 Distribution of countries/regions and institutions

#### 3.5.1 Countries/regions

**Fig. 6:**
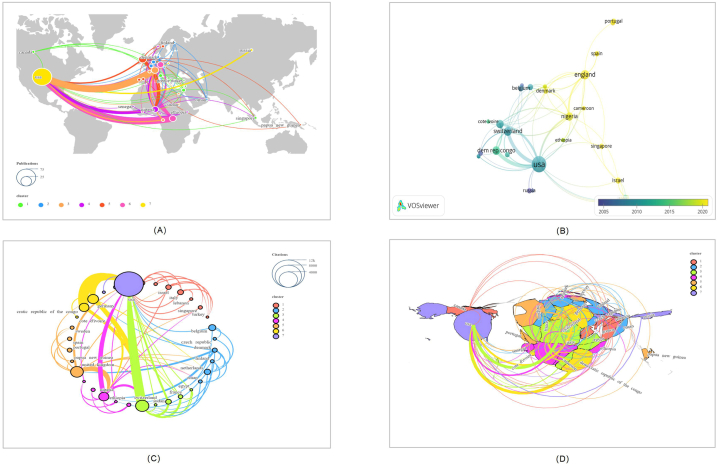
Co-citation networks among countries and regions in the top 100 papers (Listed below are: A: The total number of publications; B: the number of publications that change annually; C: the total number of citations; D: Strength of total correlation. There are more individual units on a larger graph area, and the thicker the lines between two units, the stronger the correlation.).

Research on the top 100 cited papers has been conducted by 190 institutions in 36 countries/regions. The USA contributed the most publications (N = 71), followed by ENGLAND (N = 12) and SWITZERLAND (N = 10)(Fig. 6A). Fig. 6B demonstrates the progressive publication count of articles by country/region over the years. Of these 36 countries/regions, the USA earned the most citations (N = 9177), followed by SWITZERLAND (N = 1907) and ENGLAND (N = 1751)(Fig. 6C). The 34 countries/regions were grouped into seven main clusters.In terms of the number of countries covered by national cooperative networks, the USA has established the largest with a total of 20 nations, followed by SWITZERLAND with 14 countries. It is clear from the bibliometric map that countries/regions have close relationships with each other. Collaboration among countries/regions is reflected in thicker line segments connecting nodes between countries/regions. Fig. 6D represents the total link strength of the association among country/region. ENGLAND, NIGERIA, ITALY, and SINGAPORE have released many documents over the past three years that may relate to the MPX outbreak in these countries/regions(Fig. 6B).

#### 3.5.2 Institutions

**Fig. 7:**
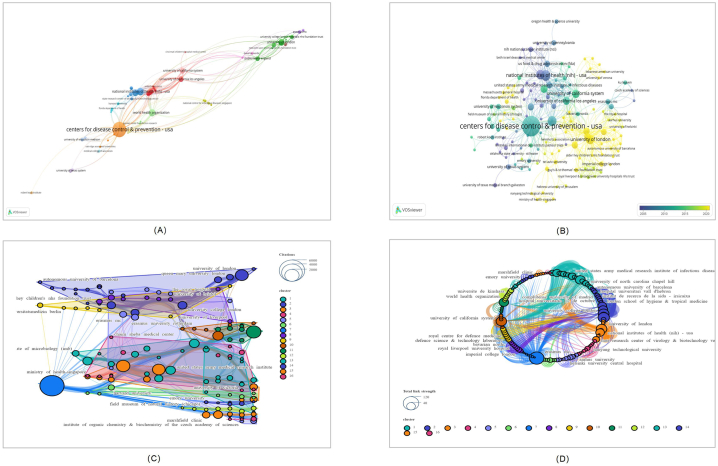
A network visualization of the institutions contributing to the 100 most cited papers (Listed below are: A: The total number of publications; B: the number of publications that change annually; C: the total number of citations; D: Strength of total correlation. There are more individual units on a larger graph area, and the thicker the lines between two units, the stronger the correlation.).

Regarding the total number of publications, the Centers for Disease Control & Prevention - USA contributed the most papers (N = 39), followed by the National Institute of Health - USA (N = 11) and World Health Organization (N = 10)(Fig. 7A). Fig. 7B reveals the evolving trend of publication volumes by institutions over the years. The most citations were received from the Centers for Disease Control & Prevention - USA (N = 5148), followed by World Health Organization (N = 1842) and National Institute of Health - USA (N = 1581)(Fig. 7C). The largest institutional cooperation network, covering 74 institutions, was formed by the Centers for Disease Control & Prevention - USA, followed by the University of London and the University of California System, with 42 and 35 institutions, respectively. A total of 142 institutions were organized into 16 major clusters, indicating that the Centers for Disease Control & Prevention - USA is a strong link between collaborations. Fig. 7D represents the total link strength of the association among institutions. In recent years, Public Health England, the University of London, the London School of Hygiene & Tropical Medicine and the University College London Hospitals NHS Foundation Trust have had the highest publication numbers (Fig. 7B).

#### 3.5.3 Research direction

According to the WOS Categories, the top 100 cited papers in MPX fall into a range of study topics, with "Immunology" (N = 34) being the most prominent research topic, followed by "Virology" (N = 16), "Infectious Diseases" (N = 15), "Multidisciplinary Sciences" (N = 7) and "Public, Environmental & Occupational Health" (N = 6).

**Fig. 8:**
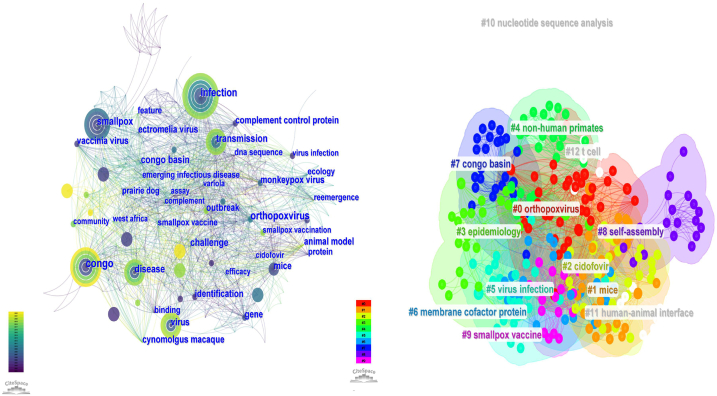
Clustering analysis and network visualization of the top 100 cited papers based on co-occurring keywords (There is a stronger correlation between graph areas with more units, and thicker lines between units with more units).

Keywordsco-occurrence, clusters and bursts; The top 100 cited papers contained 356 keywords. The most common terms were infection (N = 23), congo (N = 20), virus (N = 19), smallpox (N = 18), and transmission (N = 16). Congo (N = 20), west Africa (N = 8), and Congo Basin (N = 6) were the most frequently involved areas. Frequently referenced research animals are mice (N = 11), prairie dogs (N = 5), and cynomolgus macaque (N = 3). The most prevalent viruses are orthopoxvirus (N = 10), vaccinia virus (N = 8), monkeypox (N = 6), cowpox (N = 5), and ectromelia virus (N = 3). During the transmission of the monkeypox epidemic, the terms that people pay close attention to include infection (N = 23), transmission (N = 16), identification (N = 9), reemergence (N = 7), and outbreak (N = 6). This is compatible with the condition of a global monkeypox epidemic in 2022. Regarding specific diseases, smallpox was the most prevalent disease (N = 18)(Fig.8A). By clustering keywords on a topic, researchers can locate relevant research content. The genesis, transmission, and breakout of MPX in the Congo and other African countries are linked to the orange cluster. Animal model studies for MPX identification, prognosis, and management are mostly included in red clustering, and these studies are based on mice research. In addition to these keywords, the 16 clusters are further divided into the following: cluster 1 (orthopoxvirus), cluster 2 (mice), cluster 3 (cidofovir), cluster 4 (epidemiology), cluster 5 (non-human primates), cluster 6 (virus infection), cluster 7 (membrane cofactor protein), cluster 8 (congo basin), cluster 9 (self-assembly) and cluster 10 (smallpox vaccine) (Table 2, Fig. 8B).

**Table 2:** Top 10 co-occurring keywords found in the 100 most cited MPX papers.

Rank	Keyword	Occurrences
1	infection	23
2	congo	20
3	virus	19
4	smallpox	18
5	transmission	16
6	mice	11
7	orthopoxvirus	10
8	identification	9
9	vaccinia virus	8
10	reemergence	7

**Fig. 9:**
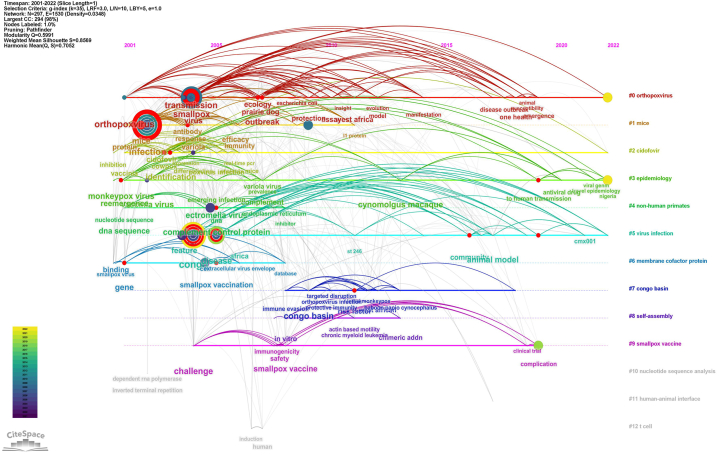
Visualization of Keyword clustering analysis of the top 100 cited papers over time.

**Fig. 10:**
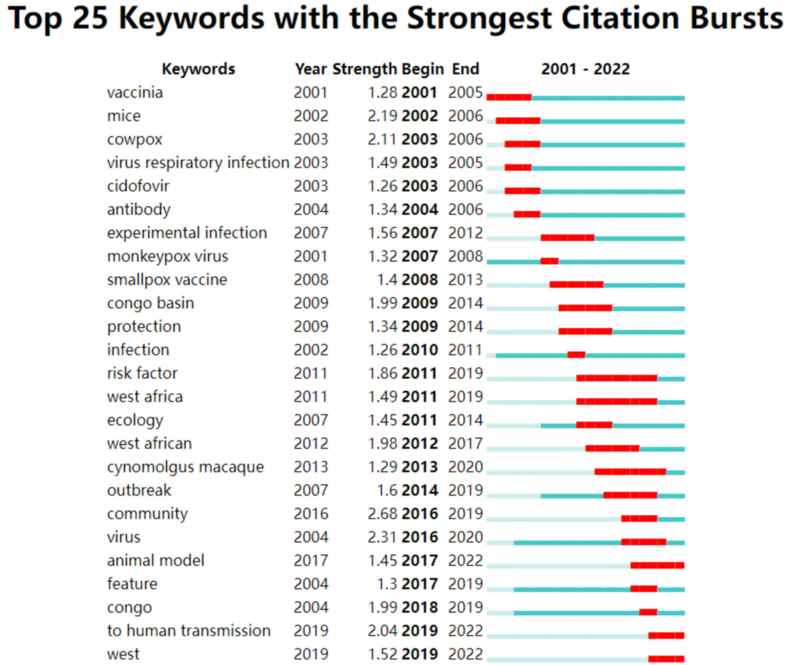
A keyword clustering analysis of the top 100 cited papers shows a year-by-year change.

Fig. 9 and Fig. 10 illustrate the trend of keyword modifications over time. Orthopoxvirus has been the center of MPX research in recent years, with researchers focusing on vaccines, safety, animal models, and pharmaceuticals. Scientists are concerned about the MPX epidemic's spread due to its reappearance and outbreak. Emerging research themes on risk and vaccination safety demonstrate that researchers worry about risk factors that could trigger widespread outbreaks. The trend of vaccinations and animal models suggests that researchers are concentrating on creating new animal models to speed up the development of novel vaccines and medications. Furthermore, recent years have centered on MPX's prevention, treatment, and danger and its related consequences.

#### 3.5.4 Co-cited articles and co-cited reference cluster analysis

**Fig. 11:**
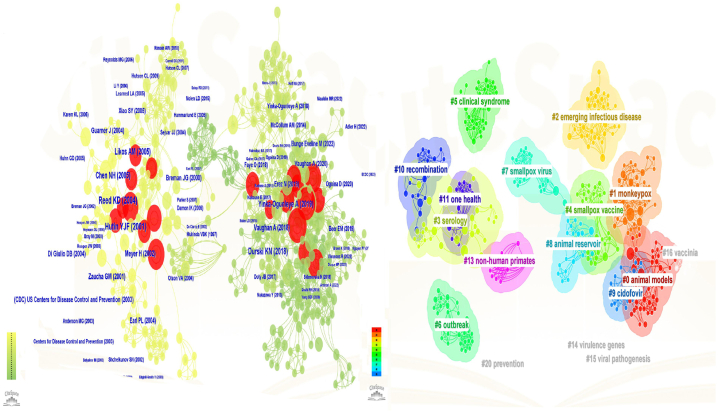
Cluster analysis of the top 100 cited papers in terms of co-cited articles and co-cited references (There is a stronger correlation between graph areas with more units, and thicker lines between units with more units).

**Fig. 12:**
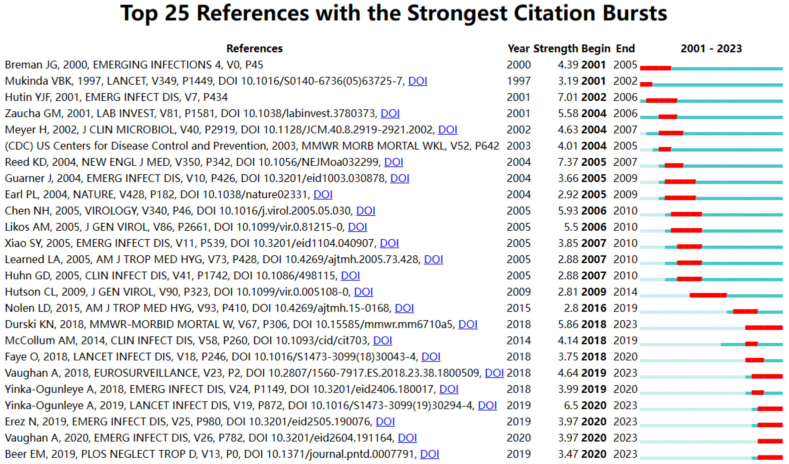
The top 25 references with the strongest citation bursts out of the top 100 cited papers.

Co-citation relationships arise when two or more articles are referenced by one or more articles simultaneously. They are widely used as a research tool to assess the degree of interconnectedness between various publications. In order to do this, we used Citespace software to create a cluster network map and to correlate co-citations. Fig. 11A displays the references that were used the most. The five hot spots on MPX research included: #1 "intramuscular," #2 "cidofovir," #3 "animal models," #4 "exportation," and #5 "west Africa," according to the cluster map (Q = 0.85, S = 0.9433). With Q > 0.3 and S > 0.5, the cluster map's cluster quality was typically acceptable. Therefore, the cluster map results in this experiment were convincing. An analysis of cited papers' co-citations and reference clusters in the top 100 papers is shown in.Fig. 11B. Citespace software was used to discover which papers were cited the most over the last ten years (Fig. 12). A list of the papers cited is shown in Table 3.

**Table 3:** Top 10 referenced papers in the top 100 cited MPX papers.

**Citation**	**Year**	**First author**	**Journal**	**Title**
19	2004	Reed KD	NEW ENGLAND JOURNAL OF MEDICINE	The Detection of Monkeypox in Humans in the Western Hemisphere
17	2001	Hutin YJF	EMERGING INFECTIOUS DISEASES	Outbreak of human monkeypox, Democratic Republic of Congo, 1996–1997
15	2018	Durski KN	MMWR-MORBIDITY AND MORTALITY WEEKLY REPORT	Emergence of Monkeypox - West and Central Africa, 1970–2017
14	2005	Chen NH	VIROLOGY	Virulence differences between monkeypox virus isolates from West Africa and the Congo basin
13	2019	Yinka-Ogunleye A	LANCET INFECTIOUS DISEASES	Outbreak of human monkeypox in Nigeria in 2017-18: a clinical and epidemiological report
13	2005	Likos AM	JOURNAL OF GENERAL VIROLOGY	A tale of two clades: monkeypox viruses
12	2001	Zaucha GM	LABORATORY INVESTIGATION	The pathology of experimental aerosolized monkeypox virus infection in cynomolgus monkeys (Macaca fascicularis)
12	2002	Meyer H	JOURNAL OF CLINICAL MICROBIOLOGY	Outbreaks of disease suspected of being due to human monkeypox virus infection in the Democratic Republic of Congo in 2001
11	2004	Guarner J	EMERGING INFECTIOUS DISEASES	Monkeypox transmission and pathogenesis in prairie dogs
11	2018	Vaughan A	EUROSURVEILLANCE	Two cases of monkeypox imported to the United Kingdom, September 2018

## 4 Discussion

In the past 20 years, researchers have made significant advances in treating MPX [26]. A similar situation to the 2017–2018 plague presents its challenges, as does the evolving epidemiology of the human MPX outbreak in May 2022.

### 4.1 General information from the top 100 cited papers

We analyzed MPX research's top 100 cited papers based on bibliometrics and visuals to reveal global trends [27]. It is possible to analyze the characteristics of publications using bibliometric analysis with the help of specific and reliable metrics [[28], [29], [30]]. Researchers in medical fields can gain valuable information about specific areas of research and future directions by assessing the top cited papers in their field [[31], [32], [33]].

Over 85% of the top 100 cited papers were original articles, which suggests a tendency for researchers to cite original research. Among the top 100 most cited papers, the one with the shortest timeframe between publication and high citation count was released in 2023. One-third of all citations were attributed to the top ten papers among the top 100. The most influential articles were written by Damon, IK, and Reynolds, MG, indicating that their field is hot right now. Reed, KD. et al. released "The Detection of Monkeypox in Humans in the Western Hemisphere" in the *NEW ENGLAND JOURNAL OF MEDICINE* in 2004, receiving the most citations [34]. This study documented the clinical symptoms, treatment, control measures, and suspected transmission routes of 11 MPX patients in Wisconsin, USA, 2003. The high link of this MPX outbreak with the West African branch was discovered using genetic targeting analyses. This is among the earliest reported of monkeypox in the Western Hemisphere, and the study emphasizes that in regions without MPX outbreaks, animal imports from high-risk areas like Africa pose the highest risk of MPX outbreaks. "Monkeypox Virus Infection in Humans across 16 Countries - April–June 2022″ by Thornhill, JP et al., published in *LANCET NEUROLOGY* in 2022. This study primarily shows that sexual engagement (particularly among gay or bisexual men) is by far the most frequently suspected source of MPX transmission [24].

Most papers were published by *EMERGING INFECTIOUS DISEASES*, and it garnered the most Co-citations. Several new journals for MPX research include *MMWR-MORBIDITY AND MORTALITY WEEKLY REPORT, EUROSURVEILLANCE*, and *LANCET NEUROLOGY*. Articles published in high-impact journals like *NEW ENGLAND JOURNAL OF MEDICINE* are more likely to be cited. Seventy-one of the top 100 referenced papers were about the US, with the Centers for Disease Control & Prevention-USA publishing the most (N = 39). This statistic shows USA's significant MPX research advancement.

## 5 Future perspectives

Over half of the top 100 cited papers used multiple techniques to intervene in MPX [[35], [36], [37], [38], [39], [40], [41], [42], [43], [44]]. This paper suggests that clinical research will continue to advance MPX treatment. Other research has examined MPX and smallpox risk factors and potential connections [[45], [46], [47]]. According to recent disclosures, researchers have been drawn to investigating the methods of identification and identification in MPXV across time [[48], [49], [50], [51]]. Several researchers have developed vaccines or tested their efficacy and safety in animals [[52], [53], [54], [55]]. The amount of study into the potential transmission animals and routes of MPX is growing yearly [56,57]. Genomic comparison is also utilized to investigate and identify MPXV risk locations [58,59]. Researchers can quickly identify MPV prognosis-improving techniques by describing early clinical signs and presentations [60,61]. Although research suggests that antiviral medicine is the best therapy, developing an effective vaccine would be more effective in lowering and preventing MPX pandemics in the long term. To understand monkeypox disease's continually changing epidemiology, international coordination for case monitoring and identification is needed.

## 6 Recommendations for clinicians

1. More than half of the field's top 100 most cited articles are about MPX interventions using several technologies. Thus, researchers must continue to study related technologies and their applications.
2. The potential risk factors and connections between MPX and Smallpox warrant attention and further research should be conducted accordingly.
3. Vaccines' development and efficacy testing, and research on animal transmission and infection routes, amongst other closely related fields, are future directions of MPX research. Thus, researchers should focus on these areas.
4. International cooperation is essential for monitoring and identifying the variola virus and understanding its changing epidemiological patterns.
5. Future research directions may include searching for biomarkers, improved imaging technologies, screening genes that pose a risk to MPX clinical treatment and conducting placebo-controlled trials.
6. MPX's pathological and physiological mechanisms, including pathogenic microorganisms' biological traits and effects on the immune system, should be studied. New treatments and ideas would result.
7. Cross-infection-related issues in MPX are not yet fully understood. Hence, research on cross-infection should be intensified to explore the influencing factors and the extent to which other disorders related to MPX affect its occurrence and spread.
8. MPX researchers should interact more and educate the public. This is best done by popularizing MPX's early symptoms and identification methods and raising public awareness and preventive measures.
9. Many MPX studies only target specific populations, like respiratory system disease patients and those with weakened immune function. It is recommended that the research scope of MPX be expanded to include different age and health status populations to enrich the research content and results of MPX.
10. Due to MPX outbreak areas changing, it is recommended to strengthen MPX monitoring and forecasting, detect future epidemic trends, and build focused prevention and emergency response plans.

## 7 Limitations

It is important to note that this study has several restrictions.However, Web of Science does not contain all previous publications, even though it is the most commonly used database for literature searches. In order to achieve an accurate analysis, we opted to use the Title Keywords search method rather than the Subject Keywords search method. There is some precision in our search results, but they could have been more comprehensive.

## 8 Conclusion

As far as we know, this is the first bibliometric evaluation of the most frequently referenced works in MPX. Our findings imply that most significant studies on MPX have been published in the *EMERGING INFECTIOUS DISEASES* and that research on Vaccine research and development may be a vital area for future study. This study area will look for biomarkers, update imaging methods, screen MPX risk genes, and conduct placebo-controlled therapeutic trials.

## Institutional review board statement

Not applicable.

## Informed consent statement

Not applicable.

## Data availability statement

Data included in article/supplementary material/referenced in article.

## CRediT authorship contribution statement

**Xuhao Li:** Data curation, Formal analysis, Methodology, Project administration, Resources, Software, Writing - original draft. **Yang Li:** Data curation, Formal analysis, Investigation, Methodology, Resources, Software. **Wenyan Yu:** Conceptualization, Data curation, Formal analysis, Methodology, Resources, Software. **Zhixia Jia:** Formal analysis, Investigation, Project administration, Software. **Jinling Li:** Methodology, Resources, Software, Supervision, Validation, Visualization. **Yuanxiang Liu:** Funding acquisition, Project administration, Supervision, Writing – review & editing. **Jiguo Yang:** Funding acquisition, Project administration, Supervision, Visualization, Writing – review & editing.

## Declaration of competing interest

The authors declare that they have no known competing financial interests or personal relationships that could have appeared to influence the work reported in this paper.

## Abbreviations

Monkeypox virus =: MPXV
Monkeypox =: MPX
The United States of America =: USA
Science Citation Index Expanded =: SCI-Expanded
Web of Science =: WOS
Title =: TI
NIH National Institute of Allergy & Infectious Diseases =: NIAID
World Health Organization =: WHO
